# Outcomes of Best-Practice Guided Digital Mental Health Interventions for Youth and Young Adults with Emerging Symptoms: Part II. A Systematic Review of User Experience Outcomes

**DOI:** 10.1007/s10567-024-00468-5

**Published:** 2024-04-18

**Authors:** Jessica E. Opie, An Vuong, Ellen T. Welsh, Timothy B. Esler, Urooj Raza Khan, Hanan Khalil

**Affiliations:** 1 The Bouverie Centre, La Trobe University, Melbourne, 3056 Australia; 2 Department of Public Health, School of Psychology and Public Health, La Trobe University, Melbourne, 3000 Australia

**Keywords:** Systematic review, Youth, Adolescent, Young adult, Online, Mental health, Youth mental health, Digital, User experience, mHealth, Adolescent health

## Abstract

Although many young people demonstrate resilience and strength, research and clinical evidence highlight an upward trend in mental health concerns among those aged 12 to 25 years. Youth-specific digital mental health interventions (DMHIs) aim to address this trend by providing timely access to mental health support for young people (12–25 years). However, there is a considerable gap in understanding young people user experiences with digital interventions. This review, co-designed with Australia’s leading mental health organization Beyond Blue, utilizes a systematic methodology to synthesize evidence on user experience in youth-oriented digital mental health interventions that are fully or partially guided. Five relevant online databases were searched for articles published from 2018 to 2023, yielding 22,482 articles for screening and 22 studies were included in the present analysis. User experience outcomes relating to satisfaction and engagement were assessed for each included intervention, with experience indicators relating to usefulness, usability, value, credibility, and desirability being examined. Elements associated with positive/negative outcomes were extracted. Elements shown to positively influence user experience included peer engagement, modern app-based delivery, asynchronous support, and personalized content. In contrast, users disliked static content, homework/log-keeping, the requirement for multiple devices, and social media integration. Asynchronous interventions showed high satisfaction but faced engagement issues, with combined asynchronous/synchronous interventions reporting better completion rates. DMHIs offer a promising platform for youth mental health support and has the potential to dramatically increase the reach of interventions through the adoption of technological and user experience best practices. While young people respond positively to many aspects of intervention modernization, such as interactive, app-based design, other concepts, such as social media integration, they need to be adopted by the field more cautiously to ensure trust and engagement.

Trial Registration CRD42023405812

**Supplementary Information:**

The online version contains supplementary material available at 10.1007/s10567-024-00468-5.

## Introduction

Recent evidence highlights an upward trend in mental health concerns among those aged 12 to 25 years (Capon et al., 2023; Twenge et al., 2019). Among many contributing factors, the COVID-19 pandemic may have intensified these challenges, with most young adults (74–87%) experiencing mental health deteriorations during the pandemic (Headspace, 2020; Radomski et al., 2023). Despite the escalating number of young adults requiring greater levels of support, access to timely mental health care is currently insufficient (McGorry et al., 2022; Mei et al., 2019). Some of the common barriers to accessing support and services include perceived stigma, privacy concerns, and poor health literacy (Amone-P’Olak et al., 2023; Renwick et al., 2022). In light of these challenges and in response to reported low levels of program and support engagement and high levels of attrition, researchers are focusing on youth-oriented digital mental health interventions (Dixon et al., 2016; Kim et al., 2019).

There is growing interest in youth-oriented digital mental health interventions (DMHIs) as a means of addressing some of the challenges associated with typical face-to-face healthcare (Babbage et al., 2022; Richardson et al., 2010; World Health Organisation, 2019). These DMHIs aim to promote engagement and adherence by providing convenient support and a positive user experience (Lattie et al., 2019; Liverpool et al., 2020). A primary benefit of these interventions is their enhanced accessibility, flexibility, and scalability (Marcu et al., 2022; Philippe et al., 2022). DMHIs also offer economic benefits, as online services are generally less costly for both client and health system alike, relative to conventional face-to-face treatments. This is attributed to, for example, an absence of overhead expenses, such as renting and cleaning a physical site, and fewer staff resources required (Ben-Zeev et al., 2021; Howard & Kaufman, 2018). Importantly, DMHIs can reduce the burden on healthcare professionals, resulting in shorter waitlist times (Gagnon et al., 2016; Haleem et al., 2021). Moreover, accessing DMHIs can overcome perceived barriers such as privacy and anonymity which might otherwise deter patients from accessing face-to-face treatment (Khanna & Carper, 2022). DMHIs can also ensure treatment integrity, providing a consistent and standardized intervention in addition to the gathering of real-time participant data (Philippe et al., 2022). Integral to their success is a thoughtful user experience design that factors in the unique needs and preferences of young users, ensuring that interfaces are intuitive, content is relatable, and engagement metrics are prioritized.

### Barriers to Online Interventions

Despite the recent growth and identified benefits of self-guided DMHIs, concerns regarding their sustained usage, appropriate utilization, and ongoing efficacy have been raised (Mehrotra et al., 2017; Opie et al., 2024a; Schueller et al., 2017). These issues of engagement may prevent users from fully benefiting from these interventions (Schueller et al., 2017). A further limitation of self-guided digital interventions is high attrition rates (Alqahtani & Orji, 2019; Karyotaki et al., 2015). There is currently a limited understanding of the factors contributing to such intervention attrition and specifically understanding how these retention rates can be improved (Alqahtani & Orji, 2019), though interface ease of use has been identified as a potential barrier (Andrews et al., 2018; Nielsen, 2012).

Individual factors, such as motivation and capability, can influence intervention engagement; however, this has not been extensively studied (Cross et al., 2022). Challenges such as low digital literacy, negative prior user experience, and costs associated with internet or program access can deter users. Other considerations include data security and privacy concerns associated with DMHIs, including the storage and sharing of personal data and risk management associated with distant, independent access (Galvin & DeMuro, 2020; Wykes et al., 2019).

Specific limitations for youth also exist, relating to intervention suitability, usability, and acceptability (Balcombe & De Leo, 2023; Bergin et al., 2020; Liverpool et al., 2020). For example, youth-specific DMHIs are recommended only if specific content and design requirements are met, such as the inclusion of videos, minimal text, and intervention personalization (Liverpool et al., 2020). Therefore, analysis of clinical or standardized outcomes alone may not be sufficient. Exploring user’s experiences and perspectives may inform the re-design and improvements of an online intervention, with the purpose of improving clinical outcomes through sustained engagement.

User experience outcomes tell us about user’s engagement with, and experience of, an intervention. They often include general feedback, satisfaction and acceptance ratings, and completion rates. To date, there are few standardized tools for measuring and evaluating a user’s experience of a digital intervention with reviews reporting heterogeneity in employed measures (Ng et al., 2019; Saleem et al., 2021; Shim et al., 2017; Welsh et al., 2023). When reported, studies tend to only provide summative evaluations of users’ experiences with online interventions (Inal et al., 2020). Formative evaluations instead are conducted to develop a deep understanding of user perceptions, informing the redesign and improvements of an intervention. Formative evaluations are essential for understanding the reasons why people may be more or less likely to engage and for addressing barriers, both known and unknown. In addition to more open-ended qualitative feedback, formative evaluation seeks to collect user feedback on specific key indicators of the experience that can be used for comparing different interventions or iterations. These key indicators of user experience are the focus of the present study.

### Intervention Guidance and Delivery

DMHIs can be delivered with varying levels of human interaction or support. Guided interventions involve interaction with a human support (e.g., clinician, peer) to boost engagement and offer both clinical and technical support (Heber et al., 2017; Werntz et al., 2023). The degree of guided support can vary, ranging from partially guided, with some elements intended to be completed independently, while others provide guidance for all elements. Guidance can be delivered synchronously (i.e., live human interaction; e.g., telehealth) or asynchronously (delayed human support; e.g., email, text message). Such supported interventions have been found to be more effective than non-supported, self-guided interventions (Leung et al., 2022; Schueller et al., 2017) (Garrido et al., 2019). In one study, DMHI adherence was improved through regular interaction with a trained support facilitator (Garrido et al., 2019). Similarly, Wei et al. (2020) identified that self-guided DMHIs focusing on relaxation and self-care for COVID-19 patients were beneficial for those with mild to moderate symptoms of depression and anxiety. More research is needed, however, to fully understand the impact of, and most appropriate level of human support.

### Gaps in Available Research

To our knowledge, prior systematic and scoping reviews that examined DMHIs (both guided and unguided), and associated user experience outcomes such as satisfaction, usability, engagement, and acceptability, have exclusively targeted adults with no youth-specific reviews (Balcombe & De Leo, 2023; Gan et al., 2022; Saleem et al., 2021; Villarreal-Zegarra et al., 2022). Furthermore, prior reviews lack specific recommendations about the level and amount of human guidance that optimizes the young adult’s user experience (Hollis et al., 2017; Lehtimaki et al., 2021). A recent systematic review identified that over 70% of preventative youth DMHIs failed to document user participation in their design and development process (Bergin et al., 2020). Overlooking youth end users’ perspectives via co-design, co-development, and by embedding their feedback may result in less efficacious and appealing DMHIs (Li et al., 2022). As Opie et al. (2024b) emphasized, DMHIs must be both effective and ensure a positive user experience is provided, necessitating the examination of not only socioemotional outcomes, but user experience outcomes also.

### The Current Study

To address the aforementioned gaps and limitations and build on the promise of emerging findings, this systematic review aims to (1) identify and synthesize the literature on user experience in youth-specific, guided and partially guided DMHIs and (2) identify user experience elements within DMHIs that are associated with improved experiences and outcomes for young people. The specific user experience indicators under examination will include feasibility and fidelity; user satisfaction; completion and adherence; mode of delivery; session number; and intervention content.

## Methods

We conducted a rapid systematic review to provide a timely evidence synthesis to our industry partner (Beyond Blue, Australia’s most well-known and visited mental health organization) and help them to inform policy decision making. This review followed the Joanna Briggs Institute (JBI) methodology (Aromataris & Munn, 2020) and Cochrane Rapid Review methodological recommendations (Garritty et al., 2021). Our reporting of the review adhered to the Preferred Reporting Items for Systematic Reviews and Meta-Analyses (PRISMA; Page et al., 2021). See Online Resource 1 for a complete PRISMA checklist. A protocol of the present review was prospectively registered in PROSPERO (registered: March 23, 2023; CRD42023405812).

Following good practice, the review methodology was codesigned and conducted alongside our key stakeholder Beyond Blue and several lived experience consumer and carer academics (Pollock et al., 2018). Collectively, the current review aimed to bring together academic, consumer, and mental health service skills, experiences, and voices.

### Inclusion Criteria

The Population, Intervention, Comparator, Outcome, and Study design (PICOS) framework (McKenzie et al., 2019) guided inclusion criteria eligibility (See Table 1). Only literature written in English was included. If necessary information was not reported in-text, the study was excluded.

**Table 1:** PICOS framework

Concept	Concept details
Population (P)	Youth (mean age 12–25 years, inclusive) experiencing non-acute, emerging, mild-to-moderate mental ill-health symptoms, with no existing psychiatric diagnosis (i.e., indicated populations were excluded)
Intervention (I)	Young adult-specific interventions. The scope of interventions was mental health or combination interventions that focused on mental ill-health *and* alcohol and other drugs (AOD) interventions were included. Entirely AOD interventions were excluded. Interventions were required to be evidence-based or informed and developed by a mental health expert. The intervention duration was brief, defined as intervention length ranging from 1 to 12 sessions and duration ranging from 0 to 12 months. Interventions were standardized and manualized (solely or partially); digitally delivered by any digital delivery method; and individually delivered. Intervention delivery channel could be: 1. Combination delivery (partially guided *and* partially self-guided) or 2. Entirely guided. Such guided delivery could be synchronous or asynchronous. Guidance could include support from a clinician, researcher, expert by experience, or a mix of experts. There were no theoretical framework parameters around included interventions
Comparison (C)	Studies that contained within-group data (i.e., examine differences among subjects who are in the same group) and between-group data (i.e., assess differences in how two or more groups differ) were included. For studies with between-group data, the comparison group could be any of the following: placebo, non-intervened control, group receiving an equivalent in-person program, or any other varied intervention
Outcome (O)	All studies were required to report on pre-post intervention socioemotional outcomes and post-intervention user experience outcomes
Study design (S)	Primary research from published and unpublished sources in the form of experimental and quasi-experimental were included. Case control studies were also included. All included studies needed to report on pre-post program user experience data

### Types of Sources

The search was limited from 14 March 2018 to 14 February 2023 due to the rapid advancement of technological interventions. Date restrictions were also applied due to the dearth of available literature pre-2018).

### Search Strategy

We followed a three-step search strategy. An initial limited search of PsycINFO was conducted, followed by analysis of the text contained in the title and abstract, and of the index terms used to describe the article. This identified the keywords and index terms used for a second search across all the databases covered by this study. The second search was a systematic search of five electronic databases PsycINFO (Ovid), MEDLINE (Ovid), CINAHL (EBSCO), Cochrane Central Register of Controlled Trials (Central; via Cochrane Library). See Online Resource 2 for a complete search strategy (concept and terms) of all included databases. The third search step was an examination of additional search databases. This included searching grey literature, identifying dissertations and theses via ProQuest Dissertations and Theses. Global Trial registries were also searched to identify ongoing studies or complete but unpublished studies, these included the Australian New Zealand Clinical Trial Register (www.anzctr.org.au) and www.ClinicalTrials.gov. The first 20 pages of Google were also searched. See Online Resource 3 for a complete grey literature search strategy. Finally, to ensure a comprehensive search was conducted, reference lists of all eligible studies and pertinent systematic reviews were manually searched to identify further studies that met inclusion criteria. Authors were not contacted for missing data. This is the same search strategy used for the first part of this study series, focusing on socioemotional outcomes of digital mental health interventions.

#### Study Screening and Selection

All records were imported to Endnote (2020) where duplicates were removed. Remaining studies were imported in Covidence (Veritas Health Innovation, 2020) and were screened at title and abstract level by three reviewers (JO, AV, HK). Studies were then screened at full-text level. At both title and abstract, and full-text, 75% of records were double screened.

#### Data Extraction

Data extraction was completed by three independent reviewers (JO, AV, HK) with disagreements resolved through conferencing. Data from each full-text article was charted by one reviewer and checked by a second independent reviewer. Data was extracted into a priori standardized data extraction forms, consistent with Tables 3 and 4.

### Quality Assessment

All studies were appraised using the Quality Assessment Tool for Quantitative Studies (EPHPP, 2010). Quality appraisal checklist response options were ‘yes,’ ‘no,’ ‘unclear,’ or ‘not applicable.’ Grey literature was critically assessed using the Authority, Accuracy, Coverage, Objectivity, Date, and Significance (AACODS) checklist (Tyndall, 2010). Studies were subsequently grouped into low risk (> 75% of quality criteria met), moderate risk (> 50% of quality criteria met), or high risk of bias (< 50% of quality criteria met). An a priori decision was made not to exclude studies based on quality. One author assessed study quality for all the papers, and a second author independently assessed the study quality of 25% of the papers (inter-rater reliability = 75% agreement). All disagreements were resolved through conferencing.

### Synthesis

Data were extracted from each study relating to the included population, the intervention, and intervention user experience elements reported on. To identify socioemotional outcome efficacy and user experience outcomes, we collated and categorized the extracted intervention characteristics and outcomes into a finite set of top-level elements to facilitate synthesis (Morville, 2014). Due to data heterogeneity, a meta-analysis was not feasible, with results instead being collated and tabulated following categorization, and results were reported narratively.

### Intervention User Experience Outcomes

As recommended by Morville (2014), we aimed to categorize the findings into seven user experience quality factors or measures: useful, usable, findable, credible, desirable, accessible, and valuable, as shown in Table 2. Considering the substantial amount of heterogeneity in the reporting of different user experiences in different studies, mapping results extracted from each study to this well-defined set of factors enabled for synthesis. However, several of these user experience elements were excluded due to lack of data. Specifically, no study reported on the *findable* element and very limited data reported on the *desirable* and *accessible* elements. We also reported on user experience sub-elements of these factors. Table 3 provides population and intervention information for each included study, grouped by delivery method and Table 4 provides a summary of extracted user experience assessments from each study.

**Table 2:** User experience outcome categories used for synthesizing extracted study data

Element	Sub-element
Useful	Usefulness Acceptability Helpful
Usable	Usage/completion Attrition/adherence Engagement
Findable	–
Credible	Safety/privacy
Desirable	–
Accessible	–
Valuable	User satisfaction

**Table 3:**
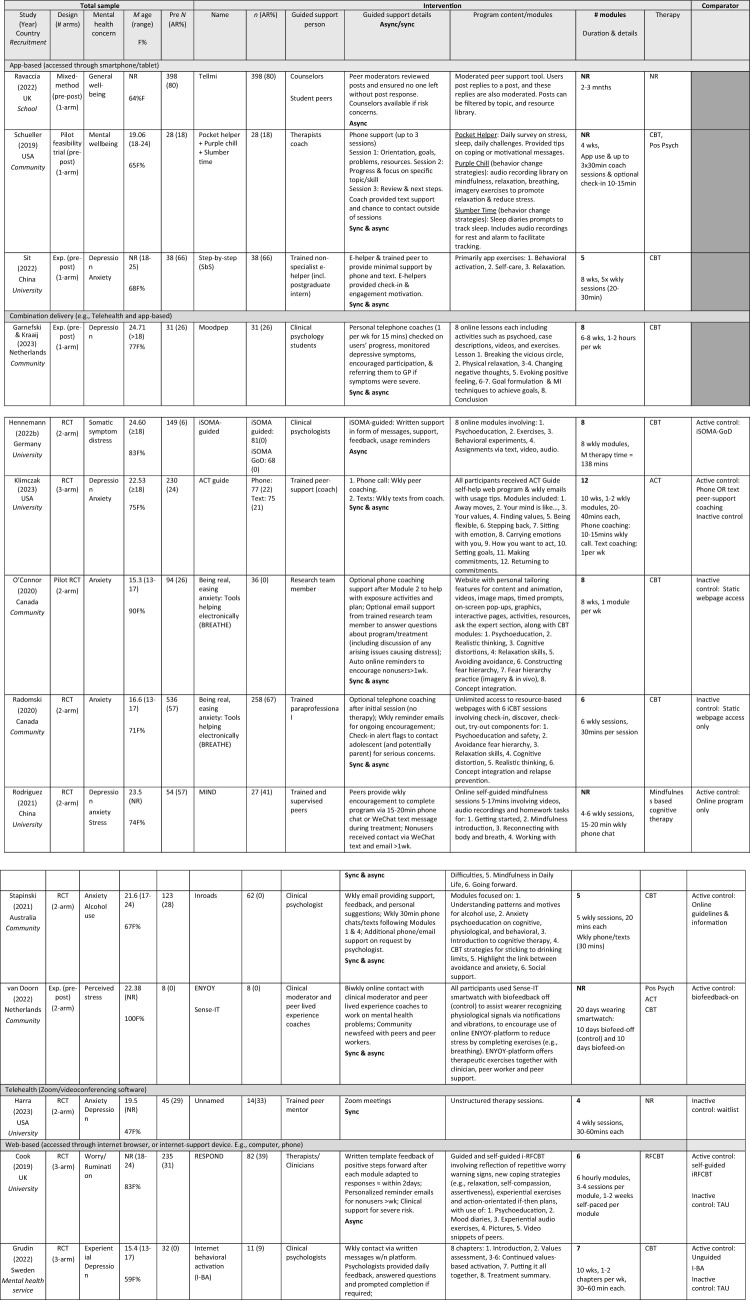

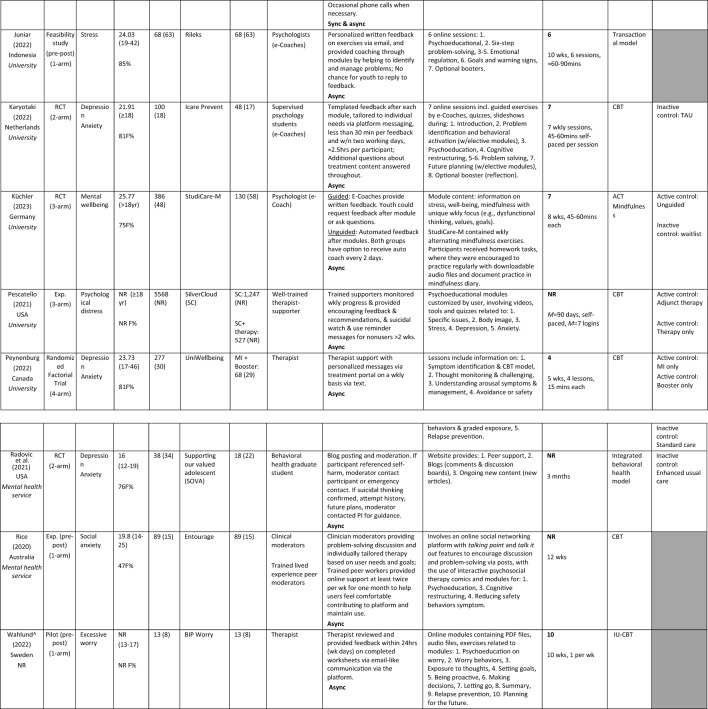
Study characteristics

^a^Unpublished thesis

*ACT* Acceptance Commitment Therapy, *Active control* Alternative intervention received, *App* Application, *AR* Attrition Rate, *Async* Asynchronous, *Auto* Automated, *Biofeed* Biofeedback, *Biwkly* Biweekly, *CBT* Cognitive Behavioral Therapy, *Exp* Experimental, *F* Female, *GoD* Guidance on Demand, *i-BA* Internet-based Behavioral Activation, *iCBT* Internet-Based Cognitive Behavioral Therapy, *Inactive control* No intervention received, *Incl* Includes/Including, *iRFCBT* Internet-based Rumination-Focused Cognitive Behavioral Therapy, *IU* Intolerance of Uncertainty, *M* Mean, *Min/s* Minute/s, *MI* Motivational Interviewing, *N* Sample size, *n* subsample size, *NR* Not Reported, *Pos Psych* Positive Psychology, *RCT* Randomized Controlled Trial, *RFCBT* Rumination-Focused Cognitive Behavioral Therapy, *Sync* Synchronous, *TAU* Treatment As Usual, *Wk* week, *Wkly* Weekly, *W/* With, *W/n* Within, Gray shading—Comparator not included in study

**Table 4:** Key user experience outcomes of included studies

Study (year) Intervention description	Synchronous guided intervention delivery				
	Outcome (measure)	Key findings			
		Feasibility and fidelity	User satisfaction	Completion and adherence	Other measures**
Harra and Vargas, (2023)* Trained peer mentor consultation over Zoom weekly for mild to moderate symptoms of anxiety and/or depression management	1.Completion	NR	21 (61.8%) appreciated opportunity to express feelings to non-judgmental listener. 4 (11.8%) enjoyed being connecting with another. 5 (14.7%) said intervention helped to learn about self and reflect	9 (64.3%) of intervention completed all four mentoring sessions, 3 (21.4%) completed 3, 2 (14.3%) complete 2	Efficacy = Yes Efficient = NR

Study (year) Intervention description	Asynchronous guided intervention delivery				
	Outcome (measure)	Key findings			
		Feasibility and fidelity	User satisfaction	Completion and adherence	Other measures
Cook et al. (2019) RCT to test whether guided Web-based RFCBT (i-RFCBT) would prevent incidence of major depression relative to usual care	1. Completion	NR	NR	*M* completion of 3.46 modules (*SD* = 2.25). 46% compliance (completion of ≥ 4 modules)	Efficacy = Yes Efficient = NR
Hennemann et al. (2022)* RCT to compare an internet-based intervention with regular psychologist support (iSOMAguided) and identify moderators for ICBT outcomes	1. Satisfaction (acceptability (CSQ-8)) 2. Completion 3. Negative effects [safety] (INEP)	NR	In both groups (internet-based intervention with regular psychologist support), 80% reported high intervention satisfaction. (iSOMA-guided: *M* = 25.57, *SD* = 4.64; iSOMAGoD: *M* = 24.12, *SD* 5.20) and did not differ significantly between group	Digital intervention completion rate was high. Participants in the iSOMA-guided group completed significantly more intervention modules on average (*M* = 5.22, *SD* = 2.40), compared to the iSOMA-GoD group (*M* = 4.09, *SD* = 2.75), *t*(134.17) = 0.69, *p* = 0.009, *d* = 0.44)	Negative effects: 18% reported one unwanted side effect of treatment (e.g., stigmatization; financial concerns; feeling dependent on partner; difficulty making decisions; longer phases of feeling bad). The frequency of negative treatment effects did not differ significantly between groups (iSOMA-guided: 10/67, 14.9%; iSOMAGoD: 11/51, 21.6%), *X*^*2*^(1) = 0.87, *p* = .350 Efficacy = Yes Efficient = NR
Juniar et al. (2022) To assess the feasibility, acceptability, usability, and efficacy of the Rileks web-based stress management intervention, as part of the preliminary version	1. Feasibility (SUS) 2. Satisfaction (CSQ-8)	Rileks is potentially feasible. The SUS mean score was 62.80 (*SD* = 14.74) for usability, which was lower than expected, with the lowest score for the learnability item (*M* = 2.88, *SD* = 1.27)	Rated as generally satisfactory	NR	Efficacy = Yes Efficient = NR
Karyotaki et al. (2022) RCT to examine effectiveness of a guided web-based transdiagnostic individually tailored iCBT	1. Satisfaction with treatment (CSQ-8) 2. Usage	NR	72% (*SD* = 7.6%) rate of satisfaction with the intervention	Participants completed approximately half of the main 7 sessions of the iCBT intervention (55%)	Efficacy = Yes Efficient = NR
Küchler et al. (2023)* RCT to evaluate the effectiveness and adherence of a revised internet- and mobile-based intervention, StudiCare-M, in an unguided and a fully guided format for college students	1. Satisfaction (CSQ-8; CEQ) 2. Negative experiences (INEP)	NR	NS differences in intervention satisfaction between UG and GoD groups	GoD participants showed significantly greater follow-up adherence after 6-months	Treatment credibility was moderate to high *M* = 20.36 (*SD* = 3.78; range 0–27) with treatment expectancy lower *M* = 18.15 (*SD* = 4.04; range 0–27). Negative experiences associated with content and e-coaching were reported more frequently with minor to moderate intensity (*t2*: *n* = 27(UG), *n* = 9(GoD); *t3*: *n* = 18(UG), *n* = 15(GoD). The most frequently reported negative experiences were “I felt forced by the StudiCare training or the e-coach to do exercises that I really didn’t want to do at all.” (*n* = 43) and “By participating in StudiCare training, I spend too much time in front of the computer and neglect my hobbies and social contacts.” (*n* = 19) Efficacy = No Efficient = NR
Pescatello et al. (2021)* To determine whether an internet-delivered psychotherapy (SilverCloud) had comparable outcomes to psychotherapy in routine care	1. Use	NR	NR	Mean intervention length 89.64 days (*SD* = 67.87; range = 4–475) for SC-ONLY, 96.09 days (*SD* = 133.44; range 3–1036) for SC + TX. On average, SC-ONLY and SC + TX participants used 15% of DHMI or viewed an average of 16.27 pages (*SD* = 17.72; Range 0–101) and used an average of 3.88 tools (*SD* = 4.98; Range 0–41)	Efficacy = Yes Efficient = NR
Peynenburg et al. (2022) To examine effects of including pretreatment MI and a self-guided booster (UniWellbeing) offered 1-month after transdiagnostic iCBT for postsecondary students	1. Satisfaction (TSQ)	NR	High satisfaction (82.3% (158/193) with participants reporting they were ‘satisfied’ or ‘very satisfied’ with treatment. 76.2% (147/193) reported confidence in their ability to manage their symptoms ‘increased’ or ‘greatly increased’ post. NS differences between intervention group on any treatment satisfaction measures *(p* = *0.*37–0.83)	NR	Efficacy = Yes Efficient = NR
Radovic et al. (2021) RCT to pilot a peer support website intervention for adolescents (SOVA) with enhanced usual care (EUC) for depression or anxiety symptoms	1. Accessibility	NR	NR	There was limited access to the website due to forgetting on not having time. 50% of adolescents reported they would forget to access the website	Efficacy = Yes Efficient = NR
Ravaccia et al. (2022) To assess the impact of using MeeToo on young people and reasons for these impacts for general wellbeing	1. Usage	NR	NR	At T1, 50% of youth had just started and 37% had been using MeToo for = > 1 mnth. At T2, 54% had been using MeToo for = > 1 month and 31% had just started	Efficacy = Yes Efficient = NR
Rice et al. (2020) To pilot a novel digital intervention (Entourage) for young people with prominent social anxiety symptoms, with a particular focus on the engagement of young men	1. Acceptability 2. Feasibility 3. Safety (PHQ-9; LSAS) 4. Usage	Feasibility indicators were met: Youth gave positive intervention feedback with 98.6% reporting they would recommend to intervention to another with social anxiety	Overall, 25.8% (*n* = 23) met the a priori acceptability criteria (logging on to Entourage = > 10 times over 10 wks). 60.7% (*n* = 54) logged in weekly over 5 wks. At post, 74.4% said Entourage provided timely support; 62.2% said Entourage therapy content relevant to developing social anxiety symptoms control; 77.0% found Entourage at least somewhat helpful	1583 total individual system logins from participants (*M*(sample) = 17.8; *M*(male) = 19.9). high participant usage of Steps modules with 1534 completed in total (*M*(sample) = 17.2; M(male) = 14.4) with an average of 4.2 Actions completed per user (*M*(male) = 3.9). Throughout pilot there were 19 separate Talk it Out group-based problem-solving topics pitched by participants and developed into solutions, with a total of 156 interactions for these. The Talking Point feature also received substantial engagement, with 80 contributions to these discussions from participants	All participants felt safe and adequately supported by clinicians while using the intervention: On a 5-point scale from ‘not safe at all’ to ‘very safe,’ most 94.1% (*n* = 32) reported feeling safe and no participants reported feeling unsafe. No serious adverse events reported during intervention. Efficacy = Yes Efficient = NR
Wahlund (2022)^a^ Dissertation to develop and evaluate streamlined psychological interventions (IU-CBT; BIP Worry) for adolescents and adults, specifically causal mechanisms relevant in the maintenance of excessive worry	1. Completion	NR	NR	92% (12/13) followed through with online intervention. Intervention module completion rates high (average completion. 9.8/10)	Efficacy = Yes Efficient = NR

Study (year) intervention	Mixed synchronous and asynchronous guided intervention delivery				
	Outcome (measure)	Key findings			
		Feasibility & fidelity	User satisfaction	Completion and adherence	Other measures
Garnefski & Kraaij (2023) Pre-post experimental study to evaluate an online self-help program, Moodpep, that provides tools to those with emerging depressive symptoms	1. Usefulness 2. User satisfaction 3. Completion	60.9% found program ‘quite useful'. Most (56.5–73.9%) intended to continue using the techniques learned in the program.	High satisfaction with program (M = 7.65, SD = 0.88; range 6–9) and coach (M = 8.48, SD = 1.04; range 7–10). 78.3% would recommend it to others. Most valued telephone coaching (87%), with 69.6% not preferring another coaching method, though 17.4% favored video calls	23/31 (74.19%) program completion	Efficacy = Yes Efficient = NR
Grudin et al. (2022)* RCT to test the feasibility and acceptability of therapist-guided and self-guided internet-delivered BA (I-BA)	1. Treatment adherence 2. Credibility 3. Satisfaction (CSQ-8; NEQ-20)	NR	Mean treatment credibility was 14.3 (*SD* = 2.7) for therapist-guided I-BA (*n* = 11), 14.1 (*SD* = 3.9) for self-guided I-BA (*n* = 9) and 11.1 (*SD* = 3.4) for TAU (*n* = 8). Average treatment satisfaction at post-treatment was 24.7 (*SD* = 5.33) for therapist-guided I-BA (*n* = 11), 21.3 (*SD* = 6.8) for self-guided I-BA (*n* = 9) and 17.7 (*SD* = 6.3) for TAU (*n* = 10)	Mean completion of 7.5 chapters (*SD* = 1.0) for adolescents and 7.4 (*SD* = 1.3) for parents in therapist-guided I-BA, and 5.4 (*SD* = 2.5) for adolescents and 5.9 (*SD* = 2.8) for parents in self-guided I-BA. Eight adolescents (73%) and eight parents (73%) in therapist-guided I-BA, and three adolescents (30%) and four parents (40%) in self-guided I-BA had completed all eight chapters by the end of treatment. Zero participants in therapist-guided I-BA, and three in self-guided I-BA, discontinued treatment	Efficacy = Yes Efficient = NR
Klimczak et al. ( 2023)* To test the efficacy of a novel peer-support coaching model for college students using ACT Guide (Web-based self-directed program with telephone call (sync)/ with text messaging (async) /no coaching	1. Adherence	NR	NR	Using phone *and* text coaching as interventions more effective at increasing adherence to ACT Guide vs. standard ACT Guide without coaching. Control, which used ACT Guide only, and had low adherence rates. Age moderated effect of text coaching on program adherence, with older individuals showing weaker effects of text coaching on adherence (*p* = 0.025). No significant moderation effect of baseline psychological distress on adherence (*p* < 0.05)	Efficacy = Yes Efficient = NR
O’Connor et al. (2020)* RCT of self-directed internet website with 8 modules about BREATHE intervention & ad hoc phone and email support wherein research member could answer questions regarding intervention	1. Satisfaction 2. Adherence 3. Acceptability	13 (93%) indicated intervention easy to use and understood all materials; 5 (36%) said intervention was difficult to complete the homework pages each week. All liked that intervention was completed online, 11 (79%) indicating no concerns with privacy. 5 (36%) agreed the intervention should include social media component, 7 (50%) agreed intervention should be more personalized, and 8 (57%) agreed intervention should include a parent module. Common barriers to intervention completion were: difficulty completing exposure activities and remembering /finding time to complete modules, among other life commitments	Mean satisfaction score among adolescents was 28.5/40 (*SD* = 4.0), indicating modest satisfaction	13 (36%) completed all 8 modules and 2 (6%) complete no modules. Completers and noncompleters did not differ significantly in responses to ASQ screening questions *(p* = *0.*32, 0.93, 0.49, and 0.49), how they learned about the study (social media/on the web, health care provider/guidance counselor, friend, or not specified; *p* = 0.17), age *(p* = *0.*85), or baseline MASC2 T scores *(p* = *0.*44)	Efficacy = Yes Efficient = NR
Radomski et al. (2020) RCT to compare a six-session iCBT program for adolescent anxiety compared to online resources alone, BREATHE intervention	1. Usage (UEQII – study-specific measure) 2. User experience (UEQII; study-specific measure)	Experience was significantly more positive for BREATHE than control *(p* < *0.*001). BREATHE users reported DHMI design and delivery factors that challenged (e.g., time constraints and intervention support) or facilitated (e.g., demonstration videos, self-management activities) use	BREATHE users had significantly higher satisfaction and acceptability (*p* < 0.001), credibility and impact (*p* < 0.001), and core items total scores (*p* < 0.001) than control	Intervention use was low (*M* = 2.2 sessions, *SD* = 2.3; *n* = 258) and webpages (*M* = 2.1 visits, *SD* = 2.7; *n* = 278), but higher for BREATHE (median = 6.0 (1–6); 81/258) and webpage respondents (median = 2.0 (1–9; 148/278). Adherence and usage score was higher among webpage users vs BREATHE users, but this difference was NS (*p* = 0.18)	Efficacy = Yes Efficient = NR
Rodriguez et al. (2021)* RCT to investigate the efficacy of internet-based mindfulness intervention (MIND) and intervention plus peer counselor support (MIND +)	1. Adherence / completion	NR	NR	Youth in MIND + (vs. MIND) has significantly less attrition and more adherence, as indicated by greater likelihood of completing post assessments (16/27, 59% vs. 7/27, 26%; χ 2 1 = 6.1; *p* = 0.01) and higher course completion (72.6/100, 72.6% vs. 50.7/100, 50.7*%; t(*52) = 2.10; *P* = 0.04), respectively. *NS* between-group differences in daily frequency and duration of mindfulness practice	Efficacy = Yes Efficient = NR
Schueller et al. (2019) To pilot a mobile phone intervention for young adults experiencing homelessness with brief phone coaching involving up to 3 sessions over a month, text messaging and mobile mental health apps (Pocket Helper, Purple Chill, Slumber Time)	1. User satisfaction 2. Completion rates/App Use	NR	Satisfaction high, with all youth (23/23) indicating they would recommend intervention. 52% (12/23) reported being ‘very’ or ‘extremely satisfied’ with intervention. 43% (10/23) indicated intervention helpful daily tips most popular intervention element, 64% (14/22) indicated they liked them ‘quite a bit’ or ‘a lot.’ 26% (6/23) indicated liking the IntelliCare apps ‘quite a bit’ or ‘a lot.’ Coach support (11/23, 48%) and office hours (10/23, 43%) less popular than daily tips but received higher satisfaction rating than IntelliCare apps. 48% (11/23) found skills learned beneficial, 43% (10/23) regularly used skills. Intervention length deemed appropriate by most (12/23). Use of skills learned during coaching sessions significantly related to satisfaction with Pocket Helper (*r* = 0.78, *p* < 0.001) and other skills apps (*r* = 0.46, *p* = 0.03)	57% (20/35) completed all 3 phone sessions, *M* = 2.09 sessions (*SD* = 1.22). Text messages sent by youth *M* = 15.06 (*SD* = 12.62) and received *M* = 19.34 (*SD* = 12.70) messages	Efficacy = Yes Efficient = NR
Sit et al. (2022) To test the effectiveness of step-by-step behavioral activation-based mental health intervention (Step-by-Step) to address depression and anxiety symptoms	1. User satisfaction	NR	Participant mean satisfaction = 7.5 (range: 7–8), regardless of number of completed sessions (range: 3–5 sessions)	NR	Efficacy = NR Efficient = NR
Stapinski et al. (2021) Evaluate psychologist-supported, web intervention (Inroads), designed with and for emerging adults, to promote adaptive coping strategies, and prevent anxiety and alcohol use concerns from progressing to chronic, co-occurring disorders	1. Intervention usefulness	NR	2-month follow-up indicated the majority found the intervention useful or very/ extremely useful (92%), of good/very good quality (97%), and would recommend it to others (92%)	39% of Inroads group completed all five modules, 51% completed = > 3 modules, 77% completed = > one module. There was a dose effect, with symptom change from baseline to follow/u on all outcomes increasing with completion of more online module	Efficacy = Yes Efficient = NR
van Doorn et al. (2022) Evaluate Sense-IT smartwatch with ENYOY-platform in reducing mental health complaints and increasing awareness of physiological parameters	1. Friendliness/Usability 2. Acceptance (Health-ITUES)	NR	Intervention found to be acceptable with moderate usability. Overall HI *M* = 3.69 (0.30); HI score impact *M* = 3.93 (0.43); HI score perceived usefulness *M* = 3.71 (0.42); HI score ease of use *M* = 3.63 (0.46); HI score user control *M* = 3.29 (0.74)	NR	Efficacy = Yes Efficient = NR

Effects: *t* T-test value, *p* Significance Value, *r* Correlation, *M* Mean, *NS* Non-Significant, *SD* Standard Deviation

Acronyms: *ACT* Acceptance Commitment Therapy, *CBT* Cognitive Behavioral Therapy, *GoD* Guidance on Demand, *I-BA* Internet-Based Behavioral Activation, *iACT* Internet-based Acceptance and Commitment Therapy, *iCBT* Internet-based Cognitive Behavioral Therapy, *MI* Motivational interviewing, *NR* Not reported, *RCT* Randomized Controlled Trial, *RFCBT* Rumination-Focused Cognitive Behavioral Therapy, *UG* Unguided, *SC* SilverCloud, *TAU* Treatment As Usual, *TX* Treatment Only, *t1* Time 1, *t2* Time 2, *t3* Time 3

Measures: *CSQ-8* Client Satisfaction Questionnaire, *CEQ* Credibility Expectancy Questionnaire, *Health-ITUES/HI* Health Information Technology Usability Evaluation Model, *INEP* Inventory for the Assessment of Negative Effects of Psychotherapy, *LSAS* Life Skills Assessment Scale, *MASC2* Multidimensional Anxiety Scale for Children—2nd Version, *NEQ-20* Negative Effects Questionnaire, *PHQ-9* Patient Health Questionnaire—9 item, *SUS* System Usability Scale, *UEQII* User Experience Questionnaire for Internet-Based Interventions, *TSQ* Treatment Satisfaction Questionnaire

^a^Unpublished thesis

^**^When users reported digital mental health intervention ability to produce a desired or intended result, efficacy is marked yes. When users reported achieving maximum productivity with minimum wasted effort, efficient is marked yes

^*^Includes comparison data

Each user experience element extracted from a study was identified as either positive or negative. This was achieved by using statistic data present in the study if its directionality was apparent (for example, 93% of participants indicated that the intervention was easy to use”). In other cases, the authors’ interpretation of collected results and comparison to provided baselines was used (for example, “the measured rate of intervention acceptance was higher than reference interventions”).

## Results

### Study Selection

**Fig. 1:**
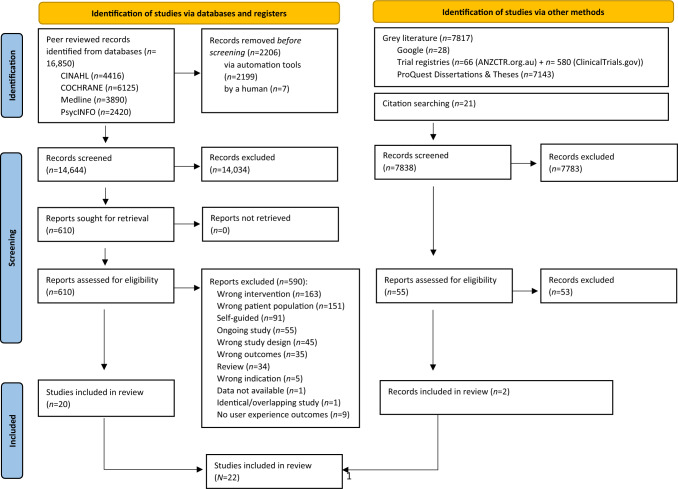
PRISMA diagram of the phases of the review process and record selection

The systematic literature search yielded 22,482 records (after removal of duplicates), of which 22,450 records were excluded at title/abstract (*n* = 21,817) and full-text level (*n* = 633). Double-screening at title and abstract resulted in inter-rater reliability (IRR) for published literature of 96% (*κ* = 0.43) and unpublished literature of 98% (*κ* = 0.45). At full-text screening, IRR was 98% (*κ* = 0.74) for published literature and 92.31% (*κ* = 0.75) for unpublished literature. A total of 31 quantitative primary studies were included in the present review (part I and part II). However, only 22 studies reported on user’s experience outcome. Hence this review will only focus on those studies. A more detailed explanation of the results of the 32 studies is provided in (Opie et al., 2024a, 2024b, this Special Issue). Figure 1 details the results at each stage of study selection and reasons for exclusion.

### Study Quality Assessment

Overall, the quality of included published studies was moderate (*n* = 12, 57%); with some of high quality (*n* = 5, 24%) and the remaining of low quality (*n* = 4; 19%). The quality of included grey literature (*n* = 1; Wahlund, 2022) was weak (i.e., high risk of bias). See Online Resource 4 for a visual and tabular representation of study quality.

### Study Characteristics

Table 3 provides a detailed description of included studies. Most studies were published studies (*n* = 21) and one was an unpublished dissertation (Wahlund, 2022). Study year ranged from 2019 to 2023, with a steady increase in the number of studies published per year.

All included studies reported on pre-post intervention outcomes, with nine studies including additional follow-up assessments. Included studies predominantly followed a RCT study design (*n* = 12, 55%), with seven single pre-post experimental studies (32%). Ten (45%) of the studies included a single comparison group (active = 5; inactive = 5), while five studies (23%) included two or more comparison groups which comprised of inactive and active controls.

Two studies reported on diverse populations. Schueller et al. (2019) included a sample of young people experiencing homelessness that were gender diverse or questioning. The intervention sample in Radovic et al. (2021) unintentionally included approximately one third (*n* = 6/20) of individuals who did not identify as male or female. Out of the 22 studies included, only 23% (*n* = 5) reported on gender diverse communities (e.g., non-binary) and/or sexual orientation. No study focused specifically on under-resourced communities or socioeconomics.

Studies were most commonly from the United States (*n* = 5, 23%), Canada (*n* = 3, 14%), and Netherlands (*n* = 3, 14%). Two studies were from Australia, China, Germany, Sweden, United Kingdom (9%, respectively), while one study was from Indonesia (5%).

### Participant Characteristics

The included study sample size was highly variable, ranging from 8 to 5568 participants, with a mean sample size of 389. Excluding studies that did not report sample age range (*n* = 5), the mean participant age was 20.97 years (range: 12–46). Six studies included only participants aged ≥ 18 years. Study participants were predominantly female, with a mean of 73.40% female participants across studies. All participants displayed emerging subclinical symptomatology.

### Intervention Characteristics

We identified 22 unique brief digital mental health interventions that are guided [entirely or partially; i.e., ACT guide; BREATHE (6-module version); BREATHE (8-module version); BIP Worry; Entourage; ENJOY + Sense-It; ICare Prevent; Inroads; I-BA; iSOMA; Tellmi; MIND; Moodpep; Pocket helper + Purple Chill + Slumber time; RESPOND; Rileks; SilverCloud; Step-by-step; StudiCare-M; SOVA; UniWellbeing; Unnamed (*n* = 1)]. These were considered brief interventions as these included less than 12 sessions.

Intervention participation length ranged from 20 days to 12 weeks (*M* = 7.60 weeks). The average number of modules per intervention was 6.87 (range: 4–12, *n* = 22), and the average number of modules intended to be completed per week of the intervention was 1.60 (range: 1–6, *n* = 10). Of the 22 studies, one study (Harra & Vargas, 2023; 5%) reported on guided interventions, which provided solely human support, while 21 (95%) reported on partially guided interventions that included a combination of human support and self-guided program elements. It was beyond the scope of this review to report on entirely self-guided digital programs. Technology delivery mode was mixed: 10 interventions were web-based, three mobile app-based (Ravaccia et al., 2022; Schueller et al., 2019; Sit et al., 2022; Sun et al., 2022), one via telehealth (i.e., Zoom/videoconferencing software; (Harra & Vargas, 2023), and eight via a combination of delivery methods.

Human guidance was provided via asynchronous methods in 11 studies, and via synchronous contact only in one study. A further 10 studies provided human guidance via a combination of asynchronous and synchronous methods. Mental health professionals were the primary providers of guided intervention content (*n* = 8, 38%), followed by clinicians and psychology students together (*n* = 5, 24%), and researchers [*n* = 1, 5% (O’Connor et al., 2020)]. Peers were the sole human support for three interventions (Harra & Vargas, 2023; Klimczak et al., 2023; Rodriguez et al., 2021). Together, peers and clinicians delivered guidance on two interventions (Rice et al., 2020; van Doorn et al., 2022), while researchers and students together delivered one intervention (Karyotaki et al., 2022). Paraprofessionals provided guidance on one intervention (Radomski et al., 2020) while clinical psychology students provided guidance in another intervention (Garnefski & Kraaij, 2023).

#### Delivery Method and Intervention Guidance

Web-delivery was the most frequent delivery method for asynchronous interventions (*n* = 9) followed by app-based interventions (*n* = 1; Ravaccia et al., 2022), and combination-delivered interventions (*n* = 1; Hennemann et al., 2022). Solely asynchronously guided support was provided through email (*n* = 1; Juniar et al., 2022), SMS/text messages (*n* = 1; Peynenburg et al., 2022), and other messaging functions built in to the intervention platform, such as chat functions (*n* = 7). Partially asynchronous guided support (*n* = 10) was provided through a mix of messaging (within platform or SMS) and phone calls (*n* = 6), emails and phone calls/text (*n* = 1; Stapinski et al., 2021), phone calls only (Radomski et al., 2020), emails (O’Connor et al., 2020), and other channels such as online support and community newsfeeds (*n* = 1; van Doorn et al., 2022).

Eleven studies were delivered solely by an asynchronous intervention, while 10 had both asynchronous and synchronous guidance. Only one intervention was solely delivered synchronously (Harra & Vargas, 2023). Due to limited data, we reported on effectiveness findings of solely and partially asynchronously guided interventions at the aggregate level.

#### Personalization

Ten interventions provided some degree of personalized messages or individually tailored content. Interventions were individually tailored according to user’s responses to interactive activities (e.g., pre-intervention survey, multiple choice questions, short writing activities, sorting tasks; Klimczak et al., 2023; O’Connor et al., 2020; Peynenburg et al., 2022; Rice et al., 2020) or users’ needs and goals (Karyotaki et al., 2022; Rice et al., 2020; van Doorn et al., 2022). Regarding timing and frequency, personalized written feedback was provided within 2 days after session completion (Juniar et al., 2022) or on a weekly basis (Stapinski et al., 2021). In one study (Cook et al., 2019), clinicians sent personalized reminder emails if there was inactivity for more than a week, while another app allowed users to adjust the frequency and the type of notifications received (Van Doorn et al., 2022).

### Intervention User Experience Outcomes

Key user experience outcomes of included studies are presented in Table 4. Several validated measures were used for assessing the user experience (User Experience Questionnaire for Internet-based Interventions [UEQII], Radomski et al. (2020); Negative Effects Questionnaire [NEQ-20], Grudin et al. (2022); System Usability Scale [SUS], Juniar et al. (2022); Client Satisfaction Questionnaire [CSQ-8], *n* = 5; Credibility Expectancy Questionnaire [CEQ], Küchler et al. (2023); Inventory for the Assessment of Negative Effects of Psychotherapy [INEP], (Hennemann et al. 2022, Küchler et al., 2023); and Health Information Technology Usability Evaluation Model [Health-ITUES], (van Doorn et al., 2022)). Unvalidated measures were also employed in 12 studies. In order to better drawn conclusions and synthesize the various and heterogeneous measures reported in different studies, the reported measures from each study were mapped to the standardized user experience elements present in Table 2 (useful, useable, findable, credible, desirable, accessible, and valuable) As shown in Table 5, most user experience measures related to usability, satisfaction, acceptance, and helpfulness (Juniar et al., 2022; Radomski et al., 2020; Rodriguez et al., 2021; Wahlund, 2022). However, no study reported on intervention findability, and limited information was reported on desirability and accessibility-related user experience factors (Juniar et al., 2022; Küchler et al., 2023; O’Connor et al., 2020; Radomski et al., 2020; Rice et al., 2020).

**Table 5:** Common intervention elements and associated user experience outcomes

Reported evidence of positive user experience		Reported evidence of negative user experience	
User experience element	Studies	User experience element	Studies
Useful			
Usefulness			
App-based program	van Doorn et al. (2022); Overall HI *M*=3.69 (0.30) indicating moderate usability.		
Automated user notifications	van Doorn et al. (2022); Overall HI *M*=3.69 (0.30).		
Acceptability			
Mixed methods delivery (e.g., app, website)	O’Connor et al. (2020); 93% indicated intervention easy to use and understood all materials. van Doorn et al. (2022); Overall HI *M*=3.69 (0.30), indicating acceptability.	Static web-based content	Radomski et al. (2020); Experience was significantly more positive for BREATHE intervention than static web-based control *(p<0.*001).
Web-based multimedia and interactivity	Garnefski & Kraaij (2023); 60.9% found program ‘quite useful'. Most (56.5–73.9%) intended to continue applying the techniques learned in the program. Rice et al. (2020); Youth gave positive intervention feedback with 98.6% reporting they would recommend to intervention to another; 74.4% said Entourage provided timely support; 62.2% said Entourage therapy content relevant to developing social anxiety symptoms control; 77.0% found Entourage at least somewhat helpful. Radomski et al. (2020); Various design elements were reported to facilitate use (e.g., demonstration videos, self-management activities). BREATHE had significantly higher credibility and impact (*p*<0.001), and core items total scores (*p*<0.001) than control.	Integration with existing social media platforms	O’Connor et al. (2020); 5 (36%) agreed the intervention should include social media component, 7 (50%) agreed intervention should be more personalized, and 8 (57%) agreed intervention should include a parent module.
		Built-in custom social media component	Rice et al. (2020): Overall, 25.8% (*n*=23) met the a priori acceptability criteria (logging on to Entourage =>10 times over 10 wks).
Usable			
Usage/Completion			
Telehealth sessions (e.g., Zoom consultation)	Harra and Vargas (2023); 64.3% of intervention completed all four mentoring sessions.	Multiple devices required (e.g., watch, phone, laptop)	van Doorn et al. (2022); Mean system usability score (SUS) was above the cut-off score (*M*= 63.78, *SD* =10.96), indicating some usability issues. Half did not use the ENJOY platform (*M*=3.63, *SD*=0.46), indicating that the user-system interaction was not optimal.
Web-based multimedia and interactivity	Garnefski & Kraaij (2023): 23/31 (74.19%) completed program. Grudin et al. (2022); Eight adolescents (73%) and eight parents (73%) in therapist-guided I-BA, and three adolescents (30%) and four parents (40%) in self-guided I-BA had completed all eight chapters by the end of treatment. Karyotaki et al. (2022); Participants completed approximately half of the main 7 sessions of the iCBT intervention (55%). Wahlund (2022); 92% (12/13) followed through with online intervention. Intervention module completion rates high (average completion. 9.8/10). Cook et al. (2019); *M* completion of 3.46 modules (*SD=*2.25). 46% compliance (completion of ≥ 4 modules). Hennemann et al. (2022); completion rate was high. Participants in the iSOMA-guided group completed significantly more intervention modules on average (*M=*5.22, *SD=*2.40), compared to the iSOMA-GoD group (*M*=4.09, *SD=*2.75), *t*(134.17)= 0.69, *p*= 0.009, *d*=0.44). Pescatello et al. (2021); Mean intervention length 89.64 days (*SD=*67.87; range = 4–475) for SC-ONLY, 96.09 days (*SD*=133.44; range 3–1036) for SC + TX. On average, SC-ONLY and SC + TX participants used 15% of DHMI or viewed an average of 16.27 pages (*SD=*17.72; Range 0–101) and used an average of 3.88 tools (*SD*=4.98; Range 0–41).		
Combination-delivered program (asynchronous and synchronous)	Stapinski et al. (2021); 39% of Inroads group completed all five modules, 51% completed ≥ 3 modules, 77% completed ≥ one module. There was a dose effect, with symptom change from baseline to follow/u on all outcomes increasing with completion of more online module.		
App-based program	Klimczak et al.( 2023); Using phone *and* text coaching as interventions more effective at increasing adherence to ACT Guide vs. standard ACT Guide without coaching. Schueller et al. (2019); 57% (20/35) completed all 3 phone sessions, *M*=2.09 sessions (*SD=*1.22). Text messages sent by youth *M*= 15.06 (*SD=*12.62) and received *M*=19.34 (*SD*=12.70) messages. 48% (11/23) found skills learned beneficial, 43% (10/23) regularly used skills. Intervention length deemed appropriate by most (12/23).		
Attrition/Adherence			
Combination-delivered program (asynchronous and synchronous)	Klimczak et al.( 2023); Using phone *and* text coaching as interventions more effective at increasing adherence to ACT Guide vs. standard ACT Guide without coaching. Rodriguez et al. (2021); Youth in MIND+ (vs. MIND) has significantly less attrition and more adherence, as indicated by greater likelihood of completing post assessments (16/27, 59% vs. 7/27, 26%; χ 2 1=6.1; *p*=0.01) and higher course completion (72.6/100, 72.6% vs. 50.7/100, 50.7%; *t(*52=2.10; *P*=0.04), respectively.		
Web-based multimedia and interactivity	Küchler et al. (2023); GoD participants showed significantly greater follow-up adherence after 6-months.		
Engagement			
Web or app-based program	Ravaccia et al. (2022); At T1, 50% of youth had just started and 37% had been using MeToo for ≥ 1 mnth. At T2, 54% had been using MeToo for ≥ 1 month and 31% had just started. Rice et al. (2020); 1583 total individual system logins from participants (*M*(sample)=17.8; M(male)=19.9). high participant usage of Steps modules with 1534 completed in total (*M*(sample)=17.2; *M*(male)=14.4) with an average of 4.2 Actions completed per user (*M*(male)=3.9). Talking Point feature also received substantial engagement, with 80 contributions to these discussions from participants.	Lengthy program content (modules >30 mins)	Radomski et al. (2020); Intervention use was low (*M*=2.2 sessions, *SD=*2.3; *n*=258). BREATHE users reported DHMI design and delivery factors that challenged use (e.g., time constraints and intervention support). O’Connor et al. (2020); 13 (36%) completed all 8 modules and 2 (6%) complete no modules. Common barriers to intervention completion: difficulty completing exposure activities and remembering / finding time to complete modules, among other life commitments. Radovic et al. (2021); There was limited access to the website due to forgetting on not having time. 50% of adolescents reported they would forget to access the website.
Peer counselling	Harra and Vargas (2023); 64.3% of intervention completed all four mentoring sessions, 3 (21.4%) completed 3, 2 (14.3%) complete 2.		
Human- or tech-prompted group discussions	Rice et al. (2020); The Talking Point feature also received substantial engagement, with 80 contributions to these discussions from participants.		
Valuable			
User satisfaction			
Web-based multimedia and interactivity	Garnefski & Kraaij (2023); High satisfaction with program (M = 7.65, SD = 0.88; range 6–9) and coach (M = 8.48, SD = 1.04; range 7–10). 78.3% would recommend program to others. Most valued telephone coaching (87%) & most would not have preferred another coaching modality (69.6%). For some, another modality preference was video call (17.4%). Juniar et al. (2022); Rated as generally satisfactory. Hennemann et al. (2022); In both groups (internet-based intervention with regular psychologist support), 80% reported high intervention satisfaction. (iSOMA-guided: *M*=25.57, *SD=*4.64; iSOMA GoD: *M*=24.12, *SD=*5.20) and did not differ significantly between group. Karyotaki et al. (2022); 72% (*SD*=7.6%) rate of satisfaction with the intervention. Peynenburg et al. (2022); High satisfaction (82.3% (158/193) with participants reporting they were ‘satisfied’ or ‘very satisfied’ with treatment. Radomski et al. (2020); BREATHE users had significantly higher satisfaction and acceptability (*p*<0.001), than control.	Web-based program (i.e., asynchronous)	Küchler et al. (2023); NS differences in intervention satisfaction between UG and GoD groups.
Combination-delivered program (asynchronous and synchronous)	Schueller et al. (2019); Satisfaction high, with all youth (23/23) indicating they would recommend intervention. 52% (12/23) reported being ‘very’ or ‘extremely satisfied’ with intervention. O’Connor et al. (2020); Mean satisfaction score among adolescents was 28.5/40 (*SD=*4.0), indicating modest satisfaction. Sit et al. (2022); Mean satisfaction 7.5 (range: 7-8), regardless of number of completed sessions (range: 3-5 sessions).		
Telehealth sessions (e.g., Zoom consultation)	Harra and Vargas (2023); 4 (11.8%) enjoyed being connected with another. 5 (14.7%) said intervention helped to learn about self and reflect.		
Credible			
Safety/Privacy			
Telehealth sessions (e.g., Zoom consultation)	Harra and Vargas (2023): 21 (61.8%) appreciated opportunity to express feelings to non-judgmental listener “e.g., “I felt safe…”.	Web-based program (i.e., asynchronous)	Hennemann et al. (2022); 18% reported one unwanted side effect of treatment (e.g., stigmatization; financial concerns; feeling dependent on partner; difficulty making decisions; longer phases of feeling bad).
Web-based multimedia and interactivity	Grudin et al. (2022); Mean treatment credibility was 14.3 (*SD=*2.7) for therapist-guided I-BA (*n*=11), 14.1 (*SD=*3.9) for self-guided I-BA (*n*=9) and 11.1 (*SD=*3.4) for TAU (*n*=8). Küchler et al. (2023); Treatment credibility was moderate to high *M*=20.36 (*SD=*3.78; range 0–27) Rice et al. (2020); All participants felt safe and adequately supported by clinicians while using the intervention. Radomski et al. (2020); BREATHE users had significantly higher credibility and impact (*p*<0.001), and core items total scores (*p*<0.001) than control. Experience was significantly more positive for BREATHE than control; they had less concerns about privacy and trusted the information *(ps<0.*001).	Integration with existing social media platforms	O’Connor et al. (2020); 11 (79%) indicating no concerns with privacy.
Desirable			
Desirability			
Combination delivered (synchronous and asynchronous)	O’Connor et al. (2020); 13 (93%) indicated intervention easy to use and understood all materials. All liked that intervention was completed online, 11 (79%) indicating no concerns with privacy.	Homework content and log-keeping	Küchler et al. (2023); The most frequently reported negative experiences were “I felt forced by the StudiCare training or the e-coach to do exercises that I really didn’t want to do at all.” Radomski et al. (2020); It was difficult to complete the homework due to time (n=4), forgetting (n=2), and feasibility issues (n=2). Schueller et al. (2019); Participants were less favourable about the IntelliCare apps (Slumber Time and Purple Chill), which involved sleep-tracking components (6/23 or 26% indicating liking them quite a bit or a lot).
Web-based multimedia and interactivity	Rice et al. (2020); At post, 74.4% said Entourage provided timely support; 62.2% said Entourage therapy content relevant to developing social anxiety symptoms control; 77.0% found Entourage at least somewhat helpful.	Web-based delivery	Juniar et al. (2022): Learnability was a challenge (*M*=2.88, *SD*=1.27). Since web-based interventions are relatively new to participants in this country (Indonesia), they had to familiarize themselves with new technical aspects related to the intervention.

Some elements are featured under multiple user experience outcomes with different study subsets

Study-element combinations were included under an outcome only when the study demonstrated some impact on said outcome

*ACT* Acceptance Commitment Therapy, *CBT* Cognitive Behavioral Therapy, *GoD* Guidance on Demand, *HI* Health Information Technology Usability Evaluation Scale, *I-BA* Internet-Based Behavioral Activation, *iCBT* Internet-Based Cognitive Behavioral Therapy, *UG* Unguided, *SC* SilverCloud, *TAU* Treatment As Usual, *TX* Treatment Only, *t1* Time 1, *t2* Time 2, *t3* Time 3

Below, we report on intervention elements or identified factors that were common to interventions reporting positive user experience outcomes (e.g., statistically significant or moderate to high percentages (i.e., > 50–100%) relating to completion, satisfaction) and negative user experience outcomes.

As shown in Table 5, DMHIs were generally found to be more *useful* and *usable* for users when they were app-based, included automated notifications, and incorporated interactive components; and less so when using static web-based content or social media components. *Usability* was also increased when programs included telehealth calls as part of a combination-delivered approach (asynchronous and synchronous), included short modules (30 min or less), and did not require the use of multiple devices. User impressions of program *credibility* were shown to also be improved by the inclusion of telehealth consultations and reduced by the inclusion of social media components. Finally, a strong negative signal was observed in user-reported *desirability* due to the inclusion of homework and log-keeping elements.

### Delivery Method

A small number of studies delivered content via a mobile app (Ravaccia et al., 2022; Schueller et al., 2019; Sit et al., 2022), and others received feedback from participants that mobile app delivery would be favorable over web-based delivery (van Doorn et al., 2022). Static online content was associated with a negative user experience (O’Connor et al., 2020; Radomski et al., 2020) when compared with didactic online learning modules. Elements that allowed participants to engage with either their peers or other intervention participants (peer counseling and prompted group discussions) were also associated with positive user experiences, with participants reporting a greater sense of engagement and social connectedness (Harra & Vargas, 2023; Rice et al., 2020). Finally, participants of interventions that involved homework components or log/diary-keeping components commonly reported these aspects as undesirable (Karyotaki et al., 2022; Klimczak et al., 2023; Küchler et al., 2023; Radomski et al., 2020; Schueller et al., 2019).

### Asynchronous Guided and Partially Guided Interventions

See Table 6 for a breakdown of effective and poor or yet-established effectiveness data for asynchronously guided interventions. Table 7 details user experience outcomes reported for each study, aggregated by level of guidance and delivery method.

**Table 6:** Asynchronously guided intervention user experience effectiveness data

Established evidence of effectiveness	Poor/undeveloped evidence of effectiveness
Solely asynchronous	
Cook et al. (2019); Hennemann et al. (2022); Juniar et al. (2022); Karyotaki et al. (2022); Küchler et al. (2023); Peynenburg et al. (2022); Ravaccia et al. (2022); Rice et al. (2020); Wahlund (2022)	Küchler et al. (2023); Pescatello et al. (2021); Radovic et al. (2021)
Asynchronous and synchronous	
Garnefski & Kraaij (2023); Grudin et al. (2022); Klimczak et al. (2023); O’Connor et al. (2020); Radomski et al. (2020); Rodriguez et al.(2021); Schueller et al. (2019); Sit et al. (2022); Stapinski et al. (2021); van Doorn et al. (2022)	O’Connor et al. (2020); Radomski et al. (2020)

**Table 7:** Studies reporting different user experience outcomes, broken down by guidance delivery and technology delivery method

	Useful				Usable					Desirable	Valuable	Credible	Accessible
Guided delivery	Acceptability	Helpfulness	Safety	Usefulness	Adverse effects	User satisfaction	Completion & adherence	Usability	Usage				
Sync						Harra and Vargas (2023)^d^					Harra and Vargas (2023)^d^		
Async	Rice et al. (2020)^a^	Juniar et al. (2022)^a^	Rice et al. (2020)^a^		Hennemann et al. (2022)^a^ Rice et al. (2020)^a^	Hennemann et al. (2022)^a^ Juniar et al. (2022)^a^ Karyotaki et al. (2022)^a^ Küchler et al. (2023)^a^ Peynenburg et al. (2022)^a^	Cook et al. (2019)^a^ Hennemann et al. (2022)^a^ Karyotaki et al. (2022)^a^ Wahlund (2022)^a^		Pescatello et al. (2021)^a^ Ravaccia et al. (2022)^b^ Rice et al. (2020)^a^	Rice et al. (2020)^a^ Küchler et al. (2023)^a^ Juniar et al. (2022)^a^	Juniar et al. (2022)^a^ Hennemann et al. (2022)^a^ Karyotaki et al. (2022)^a^ Peynenburg et al. (2022)^a^ Küchler et al.(2023)^a^	Küchler et al. (2023)^a^	Radovic et al. (2021)^a^
Sync *&* async	O’Connor et al. (2020)^c^	Garnefski & Kraaij (2023)^c^ O’Connor et al. (2020)^c^ Rodriguez et al. (2021)^c^		Stapinski et al. (2021)^c^		Garnefski & Kraaij (2023)^c^ Grudin et al. (2022)^a^ O’Connor et al. (2020)^c^ Schueller et al. (2019)^b^ Sit et al. (2022)^b^	Garnefski & Kraaij (2023)^c^ Grudin et al. (2022)^a^ Klimczak et al.( 2023)^c^ O’Connor et al. (2020)^c^ Rodriguez et al. (2021)^c^ Schueller et al. (2019)^b^	van Doorn et al. (2022)^c^	Radomski et al. (2020)^a^	O’Connor et al. (2020)^c^ Radomski et al. (2020)^a^	Radomski et al. (2020)^a^ Schueller et al. (2019)^b^ Sit et al. (2022)^b^ O’Connor et al. (2020)^c^	Grudin et al. (2022)^a^	

Studies separated based on web delivery method (^a^Web; ^b^App-based; ^c^Combination delivery; ^d^Telehealth)

No study reported on the findable element and it was thus excluded from results

*Async* Asynchronous; *Sync* Synchronous

Among asynchronous interventions, all interventions associated with high user engagement provided its users with reminders via emails or text messages after a period of delayed engagement or inactivity (> 1 week, Cook et al., 2019; Küchler et al., 2023; Rodriquez et al., 2021; five, ten, or 20 days, Hennemann et al., 2022). Furthermore, participants reported that regular reminders (i.e., on a weekly basis) were helpful (Hennemann et al., 2022; Peynenburg et al., 2022; Radomski et al., 2020) and associated with significantly greater module completion than interventions that offered irregular reminders (Hennemann et al., 2022). Radovic et al. (2021) found that asynchronously delivered interventions without regular reminders resulted in attrition.

Positive user experience outcomes were associated with asynchronously-delivered interventions that provided motivational and encouraging written feedback (Cook et al., 2019; Karyotaki et al., 2022; Küchler et al., 2023) and personalized or individually tailored messages of support from mental health professionals (Hennemann et al., 2022; Peynenburg et al., 2022; Rice et al., 2020). Similar positive user experiences were linked to receiving timely written feedback, within 24 to 48 hours after module completion (Cook et al., 2019; Juniar et al., 2022; Karyotaki et al., 2022; Küchler et al., 2023; Wahlund, 2022) and automated weekly emails or texts with personalized recommendations (Stapinski et al., 2021). Furthermore, participants reported positive experiences when coaches regularly called to monitor their progress and used motivational interviewing to promote continued participation (Garnefski & Kraaij, 2023). Positive user experiences were also tied to interventions where clinicians adhered to standardized manuals or templates for providing written feedback (Cook et al., 2019; Juniar et al., 2022; Karyotaki et al., 2022; Küchler et al., 2023).

### Intervention Session Number and Associated Outcomes

See Table 8 for effectiveness based on number of sessions. No studies reported on interventions with only one or two sessions, while only one study included three intervention sessions (Novella et al., 2022). Given the limited number of single-session interventions, we aggregated studies with fewer sessions (3–6 sessions; *n* = 7) and compared this to those with 7 or more sessions (*n* = 8). When comparing interventions by number of sessions, we found no clear difference in user experience outcomes between studies with fewer than 6 sessions (85.71%**,** *n* = 6/7 showed effectiveness), compared to those with more sessions (> 6 sessions; 75%, *n* = 6/8 showed effectiveness).

**Table 8:** Intervention user experience outcome effectiveness separated by module/session number

	Established evidence of effectiveness	Poor or yet-established efficacy
Few sessions (≤ 6 sessions)	^a^Cook et al. (2019); ^b^Harra and Vargas, (2023); ^a^Juniar et al. (2022); ^a^Peynenburg et al. (2022); ^c^Radomski (2020); ^c^Sit et al. (2022); ^c^Stapinski et al. (2021)	^c^Radomski (2020)
More sessions (> 6 sessions)	^c^Garnefski & Kraaij (2023); ^c^Grudin et al. (2022); ^a^Hennemann et al. (2022); ^a^Karyotaki et al. (2022); ^c^Klimczak et al. ( 2023); ^a^Küchler et al. (2023); ^c^O’Connor et al. (2020); ^a^Wahlund (2022)	^a^Küchler et al. (2023); ^c^O’Connor et al. (2020)

^a^Solely asynchronous interventions

^b^Solely synchronous interventions

^c^Asynchronous and synchronous combined

## Discussion

This systematic review sought to identify and examine the available published and unpublished literature, focusing on user experience of contemporary, youth-specific digital mental health interventions (DMHIs) targeting young people with emerging mental health symptoms (i.e., indicated prevention). Emphasis of the review was placed on brief DMHIs that are in full or in part guided by a human support personnel (e.g., peer, clinician).

Findings from the present study indicate that contemporary, technology-aided content delivery methods intended for indicated youth, that provide guided or partially guided support, are beneficial. Results highlighted that a positive user experience was associated with greater integration of these modern delivery methods. We also found that engagement with either peers or other intervention participants through peer counseling and prompted group discussions was associated with positive user experiences, with participants reporting a greater sense of engagement and social connectedness following DMHI participation. This is in contrast to social media integration, which was shown to negatively impact user experience. Homework or log/diary-keeping components were also often reported as undesirable by intervention participants and associated with negative experiences. Notably, homework or log/diary-keeping activities were similarly associated with negative socioemotional impacts of DMHIs (Opie et al., 2024b; this issue). It was additionally found that guided interventions showed high satisfaction rates, whether the guidance was synchronous, asynchronous, or a mixture. However, disliked elements or areas requiring improvement were typically not explicitly reported on in the examined studies. Synchronous and asynchronous combined interventions were found to have higher completion rates than solely asynchronous guided interventions, with adherence rates varying depending on the delivery method used. Consistent with prior reviews (Garrido et al., 2019; Zhou et al., 2021), we identified that web-based interventions were the most frequent delivery methods, with 48% of all interventions using this delivery mode. This suggests that diversified digital delivery methods could be drawn upon to a greater degree, which may serve to enhance user experience outcomes and broaden reach.

We found that peer engagement enhanced user experience, in line with prior research on older cohorts (Riadi et al., 2022; Saleem et al., 2021). Strong preference for peer interaction has been similarly observed in another systematic review looking at guided and unguided DMHIs in young people (Garrido et al., 2019). Despite this, peer engagement is currently an underutilized resource in DMHIs (Naslund et al., 2020; Suresh et al., 2021). Peer engagement could be a first point of engagement before clinical contact, with benefits including problem normalization, reduced power structures, cost-effectiveness, and accessibility (McGorry et al., 2022). As those working in peer support roles have typically reached a degree of recovery and maintenance during life stages and experiences similar to potential participants (Suresh et al., 2021), this has been shown to enhance client motivation and empowerment (Fortuna et al., 2019).

In the present study we identified that positive user experience outcomes were associated with interventions that provided motivational and encouraging written feedback and personalized or individually tailored messages of support from a mental health professional, in support of the findings of a prior review (Liverpool et al., 2020). Similarly, positive user experiences were associated with the provision of timely written feedback, within 24 to 48 h of module completion and automated weekly emails providing personalized suggestions. Effective asynchronous interventions with high user engagement also provided its users with reminders via emails or text messages after a period of delayed engagement or inactivity. Furthermore, regular reminders (i.e., on a weekly basis) were found to be effective and associated with significantly greater module completion than interventions that offered irregular reminders (Hennemann et al., 2022). The importance of reminders was further implied in another study that showed that interventions without regular reminders resulted in users simply forgetting to access the intervention (Radovic et al., 2021). Although not youth-specific, this is consistent with a prior systematic review wherein guided DMHIs providing automated reminders were associated with enhanced user engagement (Borghouts et al., 2021).

In line with other DMHI reviews (Liverpool et al., 2020; Struthers et al., 2015), completion and adherence rates varied depending on the delivery methods used. DMHIs had high attrition rates, with app-based interventions having the highest attrition, despite being viewed most positively by youth. Attrition rates varied according to digital delivery method, with combination-delivered studies demonstrating the lowest rates of attrition (26.83%), followed by web-based (28%), telehealth-based (29%, *n* = 1; Harra & Vargas, 2023), and app-based interventions (54.67%). These findings align with a previous meta-analysis conducted by Garrido et al. (2019), who reported that drop-out rates exceeding 20% are frequently observed. Importantly, these rates should be considered together with program reach and accessibility. For interventions aiming to reach a large number of young people, app-based interventions may enjoy a greater level of uptake at the expense of greater attrition.

Study findings suggest that investment in contemporary modes of delivery is important for usability and acceptance among young people. This includes the ability for participants to access and engage with content, support, and community through their mobile device via social media accounts, comments sections as onboarding/engagement locations, rather than solely through the web (45%, *n* = 10). This will also allow for additional interactive, rather than static, content, and the personalization of delivered content and delivery mode based on user interactions. However, integration with social media will need to be performed thoughtfully to overcome the challenges it presents with user acceptability and credibility, as shown in Table 5. The ever-increasing importance of social media in young people’s lives mandates the integration of mental health support in these forums and the overcoming of these challenges.

With common integration of online social networks within daily lives, there are opportunities and constraints in using familiar social media patterns within mental health interventions. Early feedback suggests that utilizing existing social media platforms may not be desired by participants due to privacy concerns and social stigma surrounding mental illness. However, the establishment of within-intervention online communities is likely to assist engagement and positive outcomes, and also provides a mechanism for long-term support without clinical burden.

There are a number of trade-offs between improving user experience and optimizing the socioemotional outcomes from interventions. Of note, asynchronous guidance was associated with high user satisfaction, despite commonly appearing in interventions demonstrating fewer positive outcomes for depression (Opie et al., 2024b, this issue). It will be important to strike the right balance in creating a DMHI that is both effective, feasible, and palatable. Similarly, in the present study, app-based content delivery and communication were strongly preferred among the youth cohort despite attrition rates for app-based delivery being higher than alternatives, at 54.67%. Given the importance of both socioemotional outcomes and user experience (including adherence and uptake), intervention designers will need to consider trade-offs like this carefully.

### Strengths and Limitations

While the current review has multiple strengths including a comprehensive search strategy, only articles published in the English language were included, which may have omitted some important studies. Moreover, half (*n* = 11) of the included studies recruited participants solely from university students with prodromal mental health concerns. This raises questions about generalizability considering the differing lived experience of many youth sub-populations, who often experience mental illness at higher rates than the general aggregated youth population (Cook et al., 2019; Klimczak et al., 2023; Sit et al., 2022). Considering and validating the unique experiences of broader groups may result in greater user experience outcomes, such as engagement, adherence, safety, and acceptability. One limitation of the current study is that it may have missed including some relevant research on digital mental health interventions (DMHIs). This is because the criteria for study inclusion required that the research report on both user experience outcome *and* a socioemotional outcome. As a result, studies that focused solely on user experience outcomes without addressing socioemotional outcomes may have been excluded.

### Future Research

In the future development of guided DMHIs, the principle of *user-centered design* is key. This requires inclusion of consumer, carer, and/or intervention recommenders’ input (e.g., mental health professional) throughout all phases of the development of DMHIs. Further research should focus on improving existing DMHIs by including a peer engagement component as it is currently an underutilized resource that could be a first point of engagement before clinical contact, with benefits including problem normalization, reduced power structures, cost-effectiveness, and accessibility. Further research is also required to examine differences in user experience based on module number or DMHI length. Similarly, as there is minimal research relating to single episodic interventions, we recommend exploring single session DMHIs due to their low-cost and efficient nature. The present review identified web-based programs to be the most common intervention platform; however, there was a preference for phone-based app programs (e.g., van Doorn et al., 2022). With this, future research and development projects would ideally update the formatting of these computer-only interventions to be smart-phone friendly to better suit user lifestyles and remove engagement resistance variables. Program construction should be informed by data on app usage, youth preferences and patterns, and social media engagement of target populations when moving from computer to phone-based apps.

Further DMHI research is also required to assess the utility of current interventions for diverse populations, including culturally and linguistically diverse communities, diverse socioeconomic groups, and those based in rural or regional locations. A lack of diversity in study populations limits the generalizability of interventions, highlighting the critical necessity of tailoring programs to diverse populations to account for their unique experience and meet their unique needs. There are clear constraints to methods developed and tested with predominantly white, female university students, particularly addressing findability and engagement factors for high-risk populations in need of these interventions. Further, modifications of existing interventions or the formation of specific digital mental health interventions for diverse populations is required to enhance factors such as engagement, use, relevance, and trust. Once developed, these will require assessments of efficacy.

### Implications and Translation

With common integration of online social networks within daily lives, there are opportunities and constraints in using familiar social media patterns within mental health interventions. Early feedback suggests that utilizing existing social media platforms may not be desired by participants due to privacy concerns and social stigma surrounding mental illness. However, the establishment of within-intervention online communities is likely to assist engagement and positive outcomes and also provides a mechanism for long-term support without clinical burden.

As for the number of sessions, it was difficult to draw any conclusions regarding user’s experience and the number of sessions required for an efficacious intervention. While the most common number of therapy sessions a client will attend is one (Young et al., 2012), we did not identify a brief intervention with less than three sessions. This highlights the underexplored potential of single-session or very brief digital mental health interventions for youth that are evidence-based and grounded in science. This is a notable gap in the literature. Interventions should be data-driven and consumer-informed to enhance program uptake and engagement, which in turn will likely enhance clinical efficacy outcomes. Adjunctively, research further tells us that 75% of those who drop out of therapy, on average, after that single session are happy with that one session (Barbara-May et al., 2018; Josling & Cait, 2018; Söderquist, 2018). These results have been observed internationally in Australia, Canada, and Sweden). Importantly, we must hold in mind that these research fundings do not pertain to an online therapy context. However, to date, we do not have such data for the online therapy setting.

## Conclusion

This review highlighted several factors that are associated with positive user’s experience toward DMHIs including engagement with peers; adoption of modern, technology-aided content delivery methods; and asynchronous mode of delivery. However, while many contemporary digital modes of delivery hold promise, they also present challenges that need to be thoughtfully addressed. The future of DMHIs lies in incorporating user-centered design, prioritizing the needs and preferences of its target audience, and ensuring wise-reaching applicability by catering to diverse populations.

### Supplementary Information

Below is the link to the electronic supplementary material.

Supplementary file1 (DOCX 53 KB)
